# *Lmx1a* is essential for marginal cell differentiation and stria vascularis formation

**DOI:** 10.3389/fcell.2025.1537505

**Published:** 2025-03-05

**Authors:** Justine M. Renauld, Igor Y. Iskusnykh, Ebenezer N. Yamoah, Richard J. H. Smith, Corentin Affortit, David Z. He, Huizhan Liu, David Nichols, Judith Bouma, Mahesh K. Nayak, Xin Weng, Tianli Qin, Mai Har Sham, Victor V. Chizhikov, Bernd Fritzsch

**Affiliations:** 1 Department of Biomedical Sciences, Creighton University, Omaha, NE, United States; 2 Department of Anatomy and Neurobiology, The University of Tennessee Health Science Center, Memphis, TN, United States; 3 Department of Translational Neuroscience, College of Medicine, University of Arizona, Pheonix, AZ, United States; 4 Molecular Otolaryngology and Renal Research Laboratories, University of Iowa, Iowa City, IA, United States; 5 School of Biomedical Sciences, The Chinese University of Hong Kong, Shatin, Hong Kong SAR, China; 6 Department of Neurological Sciences, University of Nebraska Medical Center, Omaha, NE, United States

**Keywords:** *Lmx1a*, pendrin, Slc26a4, stria vascularis, spiral prominence, pigment cells

## Abstract

The transcription factor *Lmx1a* is widely expressed during early inner ear development, and mice lacking *Lmx1a* expression exhibit fusion of cochlear and vestibular hair cells and fail to form the ductus reuniens and the endolymphatic sac. *Lmx1a dreher* (*Lmx1a* ^*dr/dr*^), a recessive null mutation, results in non-functional *Lmx1a* expression, which expands from the outer sulcus to the stria vascularis and Reissner’s membrane. In the absence of *Lmx1a*, we observe a lack of proteins specific to the stria vascularis, such as BSND and KCNQ1 in marginal cells and CD44 in intermediate cells. Further analysis of the superficial epithelial cell layer at the expected stria vascularis location shows that the future intermediate cells migrate during embryonic development but subsequently disappear. Using antibodies against pendrin (*Slc26a4*) in *Lmx1a* knockout (KO) mice, we observe an expansion of pendrin expression across the stria vascularis and Reissner’s membrane. Moreover, in the absence of *Lmx1a* expression, no endocochlear potential is observed. These findings highlight the critical role of *Lmx1a* in inner ear development, particularly in the differentiation of cochlear and vestibular structures, the recruitment of pigment cells, and the expression of proteins essential for hearing and balance.

## Introduction

In mammals, a hearing (cochlea) and balance system (three semicircular canals, utricle, and saccule) are separated by the ductus reuniens that provides a connection between the cochlear basal tip with the saccule (Kopecky et al., 2012; Smith et al., 2024). The stria vascularis provides high potassium in the cochlea to generate an endocochlear potential of ∼80–100 mV (Köppl and Manley, 2018; Strepay et al., 2024; Wangemann, 2006), whereas the vestibular system does not show an elevated potential of ∼1–2 mV (Hibino et al., 2010; Wangemann, 1995). A typical formation of the stria vascularis (Bovee et al., 2024; Thulasiram et al., 2024) is essential to separate the ductus reuniens from the saccule (∼1 mV) with the basal turn of the cochlea (∼80 mV). Moreover, the narrow ductus reuniens of 0.14 mm might be blocked, resulting in Meniere’s disease (Hornibrook et al., 2021). In contrast to the narrow ductus reuniens, only three mutants have been described that fuse the basal tip with the saccule that eliminates the ductus reuniens: *Lmx1a* (knock out) KO, *n-Myc* KO, and *Irx3/5* (double knock out) DKO (Fritzsch et al., 2024; Kopecky et al., 2011; Nichols et al., 2008). An incomplete fusion of utricle and saccule is shared between *Otx1* KO mice and *Lmx1a* KO (Fritzsch et al., 2001; Nichols et al., 2008). One mutant survives beyond birth to study the function of the cochlear system and the stria vascularis in the absence of a ductus reunion: *Lmx1a* KO mice (Supplementary Figure S1).

*Lmx1a*, like *Lmx1b*, is one of the LIM-homeodomain transcription factors expressed in the inner ear (Chizhikov et al., 2021; Huang et al., 2008). Previous work have shown that an A-to-T transversion in exon 2 results in an aspartate-to-valine substitution at amino acid 44, which effectively creates null alleles for *Lmx1a*, including the dreher mutation (Chizhikov et al., 2006). Several human mutations in *LMX1A* have been associated with hearing loss, but the exact cellular mechanisms of deafness are unclear (Lee et al., 2020; Schrauwen et al., 2018; Wesdorp et al., 2018; Xiao et al., 2023). Previous research in mice showed that *Lmx1a* expression participates in mechanisms that maintain separation between the posterior crista and basal cochlear sensory epithelium (Koo et al., 2009; Nichols et al., 2008; Steffes et al., 2012). Moreover, others showed that the absence of *Lmx1a* led to the ablation of the endolymphatic sac (Nichols et al., 2008; Roux et al., 2023). The continuation of the basal turn blends with the saccule and the utricle, abolishing the ductus reuniens and the utriculosaccular foramen. During the early development of the inner ear (E8.5) and until embryonic day E16.5, *Lmx1a* remains broadly expressed in the otic non-sensory epithelium (Failli et al., 2002; Huang et al., 2008; Koo et al., 2009; Nichols et al., 2020; Nichols et al., 2008; Steffes et al., 2012). This includes the epithelium that will form the Reissner’s membrane, the marginal cell layer of the stria vascularis, and the outer spiral sulcus (Jean et al., 2023; Qin et al., 2024; Renauld et al., 2021). After E18.5, *Lmx1a* expression withdraws from Reissner’s membrane and the marginal cell layer but persists in the outer sulcus (Huang et al., 2008; Koo et al., 2009; Nichols et al., 2008).

The stria vascularis, located lateral to the organ of Corti and between the outer sulcus and Reissner’s membrane, is a complex epithelium. The stria vascularis is responsible for the specialized ionic environment in the endolymph and the positive endocochlear potential that is indispensable for hearing sensitivity (Koh et al., 2023; Strepay et al., 2024). Morphologically, the stria vascularis is formed by three main cell layers, namely, the marginal, intermediate, and basal cells. Each cell layer arises from a distinct embryonic origin, with the marginal cells originating from the otic vesicle, the intermediate cells from the neural crest cells, and the basal cells from the otic mesenchyme (Bovee et al., 2024; Renauld et al., 2022; Rose et al., 2023; Sagara et al., 1995; Steel and Barkway, 1989; Trowe et al., 2011). Although the stria vascularis is typically classified as an epithelium, it is unusual as it lacks a basal lamina underneath the basal cell layer. During development, the basal lamina beneath the marginal cells is degraded around birth to allow the interdigitation between marginal and intermediate cells (Kikuchi and Hilding, 1966; Sagara et al., 1995). Furthermore, the abundant blood capillaries in the stria vascularis possess their basilar membrane (Kikuchi and Hilding, 1966). Additionally, the stria vascularis is distinct in having tight junctions that seal both its luminal (marginal cell layer) and abluminal (basal cell layer) surfaces (Kikuchi and Hilding, 1966; Koh et al., 2023; Steel and Barkway, 1989). This tight junctional seal, which separates the stria vascularis from the rest of the inner ear, confers a highly resistive pathway essential for generating endocochlear potential for hearing (Diaz et al., 2007; Gow et al., 2004; Kitajiri et al., 2004; Koh et al., 2023; Nin et al., 2008; Qin et al., 2024; Roux et al., 2023; Souter and Forge, 1998; Wang et al., 2009; Wangemann, 2006).

Adjacent to the stria vascularis, other cells play a crucial role in maintaining inner ear fluid homeostasis. A mutation in a gene affecting the gap junctional system, essential for K^+^ recycling across the stria vascularis, results in profound hearing losses (Chan and Chang, 2014; Mei et al., 2017; Szeto et al., 2022). A recent analysis of the pendrin null mutation (*Slc26a4* ^−/−^) revealed that in the cochlea, pendrin is needed for the development of the spindle cells and the spiral ligament contains extrinsic cellular components that enable cell-to-cell communication (Koh et al., 2023). Without pendrin expression, pH regulation is altered, leading to the absence of endolymphatic potential (Kim and Wangemann, 2011; Koh et al., 2023). In addition, the loss of *SOX9* and *SOX10* genes reduces *Slc26a4* expression, resulting in endolymphatic dysregulation and hydrops (Szeto et al., 2022).

Despite its essential role in hearing, generating the endocochlear potential, promoting K^+^ recycling, and the numerous human deafness mutations resulting from the genes expressed in the stria vascularis, little is known about its molecular developmental mechanisms (Pingault et al., 2010; Ritter and Martin, 2019; Thulasiram et al., 2024). During inner ear development, the cochlea’s roof epithelium, which expresses OC90 can be divided into two populations as early as E13.5 (Hartman et al., 2015; Qin et al., 2024). One cell population is *Wnt4*-positive, which gives rise to Reissner’s membrane, and the second population is GSC-positive, representing the future marginal cell layer (Qin et al., 2024). Recent findings have shown that the absence of *Esrp1*, an RNA binding protein, expands the expression of *Otx2*, potentially through FgF9/FgFr2-IIIc signaling, leading to the replacement of the stria vascularis by Reissner’s membrane (Rohacek et al., 2017).

In this study, we show that *Lmx1a* is essential for the development of the marginal cells, and its absence leads to an expansion of pendrin-expressing cells and the replacement of the stria vascularis by the outer sulcus and spiral prominence, which contains the spindle cells. We also showed that *Lmx1a* is essential for the stria vascularis epithelium development and migrating melanoblasts’ recruitment to integrate the intermediate cell layer of the stria vascularis. The results showed that in the absence of *Lmx1a*, the endocochlear potential is abolished, explaining the previously published profound deafness in the *Lmx1a* null mutant (Steffes et al., 2012).

## Materials and methods

### Experimental animals

We bred *Lmx1a* heterozygotes [*Lmx1a* drJ, Jackson Laboratory strain #000636 (Chizhikov et al., 2006)] to generate null, heterozygote, and control mice [*Lmx1a* ^*+/+*^ (WT), *Lmx1a* ^*dr/+*^ (Het), and *Lmx1a* ^*dr/dr*^ (KO)]. According to JAX, a total of 26 mutations of *Lmx1a* are known, with more details available for 16 spontaneous mutations (https://www.informatics.jax.org/marker/MGI:1888519). Eight spontaneous mutations are among those associated with hearing and vestibular defects. We know that a transversion in exon 2 resulted in an aspartate-to-valine substitution, effectively creating null alleles for *Lmx1a* (Chizhikov et al., 2006). Mice were collected at E12.5, E13.5, E15.5, E18.5, P2, P8, P10, P14, and P21; genotyped; and fixed in 4% PFA in 0.1 M P0_4_ with 300 mM sucrose. Fixation was reduced to 0.4% PFA in 0.1 M PO_4_ with 300 mM sucrose before shipping the fixed mice to UNMC for processing. All animal procedures were approved by the Institutional Animal Care and Use Committee (IACUC) of the University of Tennessee Health Sciences Center [IACUC #15-057 and 18-037] and Creighton University (IACUC #10-35).

### Histology on whole mounts

Animals were dissected to isolate the inner ear of control and *Lmx1a* KO mice. Frozen cochlear whole mounts were blocked and permeabilized with a 30% normal goat serum solution and 0.3% Triton X-100 in 1X PBS. Primary antibodies were incubated overnight in 1% normal goat serum solution and 0.1% Triton X-100 in 1X PBS. Cochlear and vestibular samples were immunostained with anti-pendrin (1:100, #2842). Alexa Fluor 488 (Thermo Fisher Scientific Cat#A-21202, RRID: AB-141607) was used as a secondary antibody at a 1:1,000 concentration for 12 h at room temperature (Koh et al., 2023). DAPI (Life Technologies) was used to highlight the expression of all cell nuclei. We also used a recently developed antibody against pendrin [Supplementary Figure S2 (Roux et al., 2023)].

Antibodies were applied to whole mounts flattened after Reissner’s membrane was cut open. In addition, sections were taken at 50-µm thickness using a vibratome, allowing for detailed expression of DAPI and pendrin. Images were captured using a Zeiss 700 microscope at 10x (0.45 NA), 20x (0.8 NA), and 40x (1.3 NA) magnification. Imaging was processed using Zen 3.8 to generate z-stacks and single images.

### Dye tracing

Dye tracing was performed by inserting dye into the brainstem to label afferents leading to the cochlea, as described by Elliott et al. (2023) and Nichols et al. (2008).

### Histology on cryosection

Samples were rinsed with PBS thrice, then placed in 30% sucrose overnight, and embedded in OCT. Sections were performed at −20°C with a 12 µm thickness on a cryostat (Leica). Slides were dried at room temperature, then rinsed with PBS, and permeabilized with Triton X-100 1% for 10 min, followed by blocking in 10% donkey serum for 30 min at room temperature. Primary antibodies diluted in 5% serum/PBS were placed on the slides overnight at 4°C [BSND (Rabbit AB196017-1001; 1/100); CD44 (Rat MA4405; 1/250); KCNQ1 (Guinea-pig APC-022-GP; 1/200) anti-pendrin (1:100, #2842) (Renauld et al., 2021; Renauld et al., 2022)]. The primary antibodies were washed three times, with each washing lasting 5 min, in PBS, and the secondary antibodies were placed at room temperature for 1 h (Alexa anti-rabbit 488; Alexa anti-guinea-pig 488 and Alexa anti-rat 555). Phalloidin-635 (A34054; 1/1,000) was used in conjunction with the secondary antibodies. Images were taken using a Zeiss 700 confocal imaging microscope at 10x (0.45 NA), 20x (0.8 NA), and 40x (1.3 NA) magnification. Imaging was processed using Zen 3.8 to generate z-stacks and single images.

### *In situ* hybridization

Whole-mount *in situ* hybridizations were carried out according to the standard procedures (Pauley et al., 2003) using previously characterized digoxigenin-labeled riboprobes for dopachrome tautomerase (*Dct*) (Steel et al., 1992) and *Tbx18* (Trowe et al., 2011; Trowe et al., 2008). Anti-digoxigenin-AP antibody and BM Purple (Roche) were used for colorimetric signal detection. Stained tissue was embedded in soft epoxy resin, sectioned, and documented as described above. Minimizing exposure to 100% EtOH and propylene oxide during embedding is critical for preserving this stain. Hybridizations omitting probes yielded a uniformly pale background stain. Where two ears are described as being simultaneously stained, all steps in the staining procedure were conducted in the same reaction vial. Identical camera settings were used to image different samples.

### Endocochlear potential measurements

Animals were anesthetized with ketamine (16.6 mg/mL) and xylazine (2.3 mg/mL) and supplemented as needed to maintain a surgical plane of anesthesia. The core temperature was maintained at 38°C with a heating pad. An incision was made in the inferior portion of the right postauricular sulcus. The bulla was perforated, allowing for exposure of the stapedial artery and the cochlea’s basal and upper turns. A hole was made in the wall of the cochlea near the basal turn using a fine drill. A glass capillary pipette electrode (10–20 MΩ) filled with 150 mM KCl was mounted on a hydraulic micromanipulator and advanced until a stable potential was observed that did not change with increased electrode depth. The ground electrode was implanted in the dorsal neck muscles. The biological signals (filtered at 1 kHz) were amplified under current-clamp mode using an Axopatch 200B amplifier (Molecular Probe, Sunnyvale, CA) and acquired using pCLAMP 9.1 software (Molecular Probe) and Digidata 1322B. The voltage changes during entry into the endolymph were continuously recorded under the gap-free model using Clampex in the pCLAMP software package (version 9.2, Molecular Probe). The sampling frequency was 10 kHz. Data were analyzed using Clampfit and Igor Pro (WaveMetrics, Portland, OR). Six animals from each age/genotype were used.

## Results

### *Lmx1a* is required for the differentiation of the marginal cells and the formation of the stria vascularis

**FIGURE 1:**
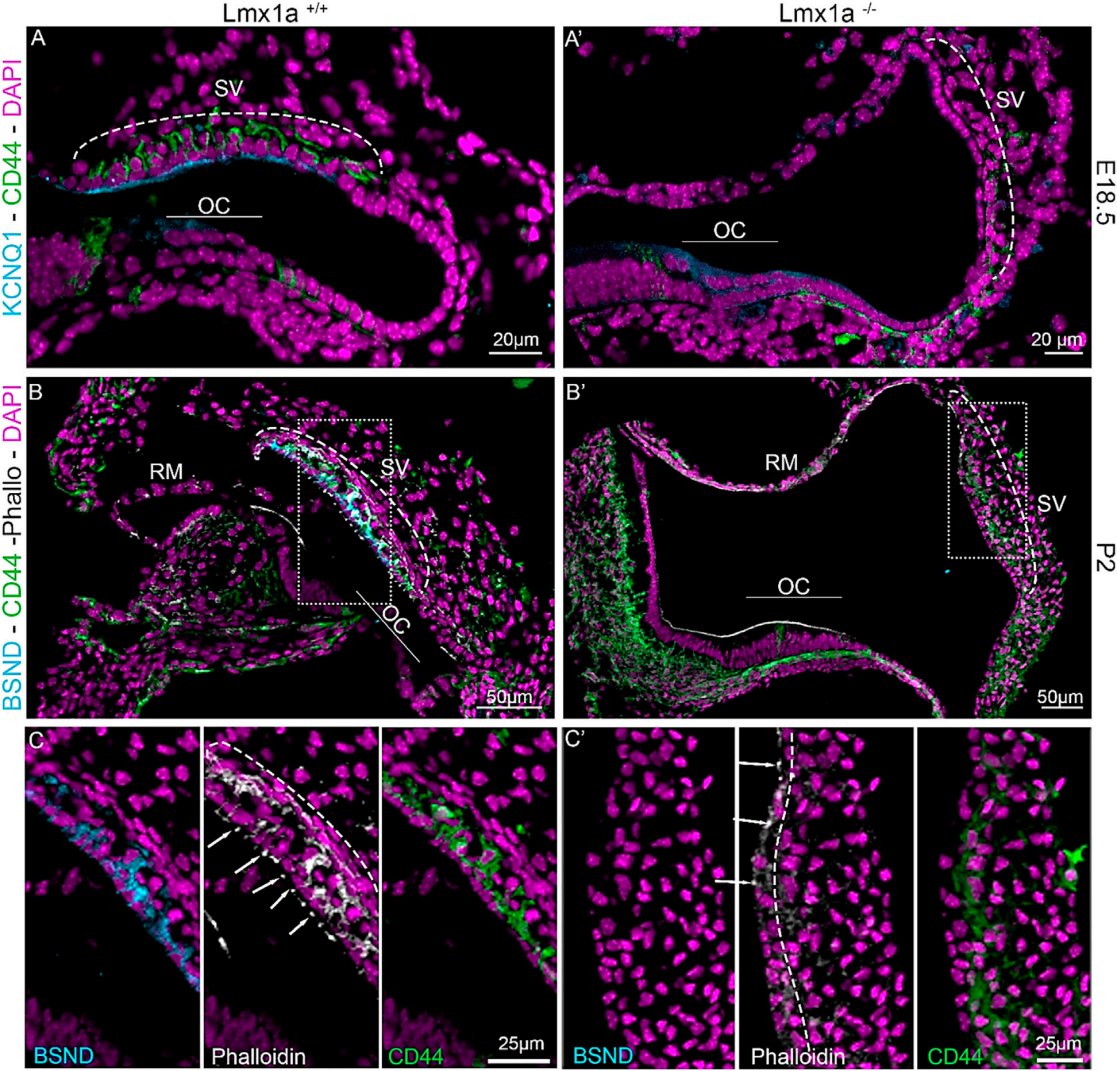
*Lmx1a* ^*−/−*^ mutant cochlea does not express marginal cell markers and fails to develop a proper stria vascularis. Immunostaining of E18.5 and P2 *Lmx1a* ^+/+^ **(A–C)** and *Lmx1a* ^−/−^ **(A′–C′)** cochlea with the white dashed rectangle magnified in inserts. Future intermediate cells labeled with CD44 (green) are on top of the cochlear duct and ingress in the lateral wall between the marginal cells labeled with Kcnq1 (cyan) in the WT cochlea **(A)**. In the *Lmx1a* ^−/−^ cochlea, at E18.5, CD44-positive cells are visible above the lateral wall but do not ingress as the marginal cell layer is absent **(A′)**. At P2, *Lmx1a* ^+/+^ cochlea showed a multilayered stria vascularis, where the first layer is labeled with BSND (cyan), another marker for marginal cells, CD44 for intermediate cells (green), and Phalloidin (white), which highlights the actin-based tight junction closing the marginal layer (arrows) and the basal layer (dashed line) of the stria vascularis **(B)**. In *Lmx1a* ^−/−^, the stria vascularis cannot be recognized without BSND labeling (cyan) and phalloidin staining on the basal side of the stria (dashed line **(C′)** insert). Some CD44-positive cells are still present but do not create the intermediate cell layer **(B′, C′)**. OC, organ of Corti; SV, stria vascularis; RM, Reissner’s membrane.

Previous publications have shown the expression of *Lmx1a* during cochlea development. *Lmx1a* is expressed as early as E8.5, and by E10.5, it is expressed in almost the entire otocyst. After initial expression, *Lmx1a* becomes more absorbed in the endolymphatic sac, which is absent in E18.5-old mice, and the lateral side of the cochlear duct (Mann et al., 2017; Nichols et al., 2020; Nichols et al., 2008). To understand the role of *Lmx1a* in the lateral wall formation, we analyzed the cochlear ducts of *Lmx1a* WT and KO at E18.5 and P2 through histological sections (Figure 1). To recognize the different cell layers of the stria vascularis, we used well-known markers such as BSND (barttin) and KCNQ1 (potassium voltage-gated channel subfamily Q member 1) for the marginal cell layer and CD44, a cell surface adhesion receptor for the intermediate cell layer (Morris et al., 2006; Rickheit et al., 2008; Rohacek et al., 2017; Sakagami et al., 1991). KCNQ1, a channel protein essential for extruding K^+^ into the endolymph and ordinarily present at the luminal surface of the marginal cells, is absent in *Lmx1a* ^−/−^ mice (Figures 1A, A′). In *Lmx1a* ^+/+^ mice, BSND labels the marginal cells and CD44 labels the intermediate cell layer. Phalloidin allows the delineation of the stria vascularis, which is rich in actin (Figures 1B, B′). In *Lmx1a* ^−/−^ mice, BSND expression is absent, and phalloidin labeling highlights a superficial epithelial layer in opposition to the three layers present in the WT (Figures 1C, C′, middle panel). The absence of landmark proteins in the marginal and intermediate cells of the stria vascularis, along with the morphology of single-cell epithelium, shows that the cellular architecture of the stria vascularis is severely compromised in *Lmx1a* ^−/−^ KO mice (Supplementary Figure S1).

### Intermediate cells’ melanoblasts migrate during development but fail to integrate into the stria vascularis without *Lmx1a*

**FIGURE 2:**
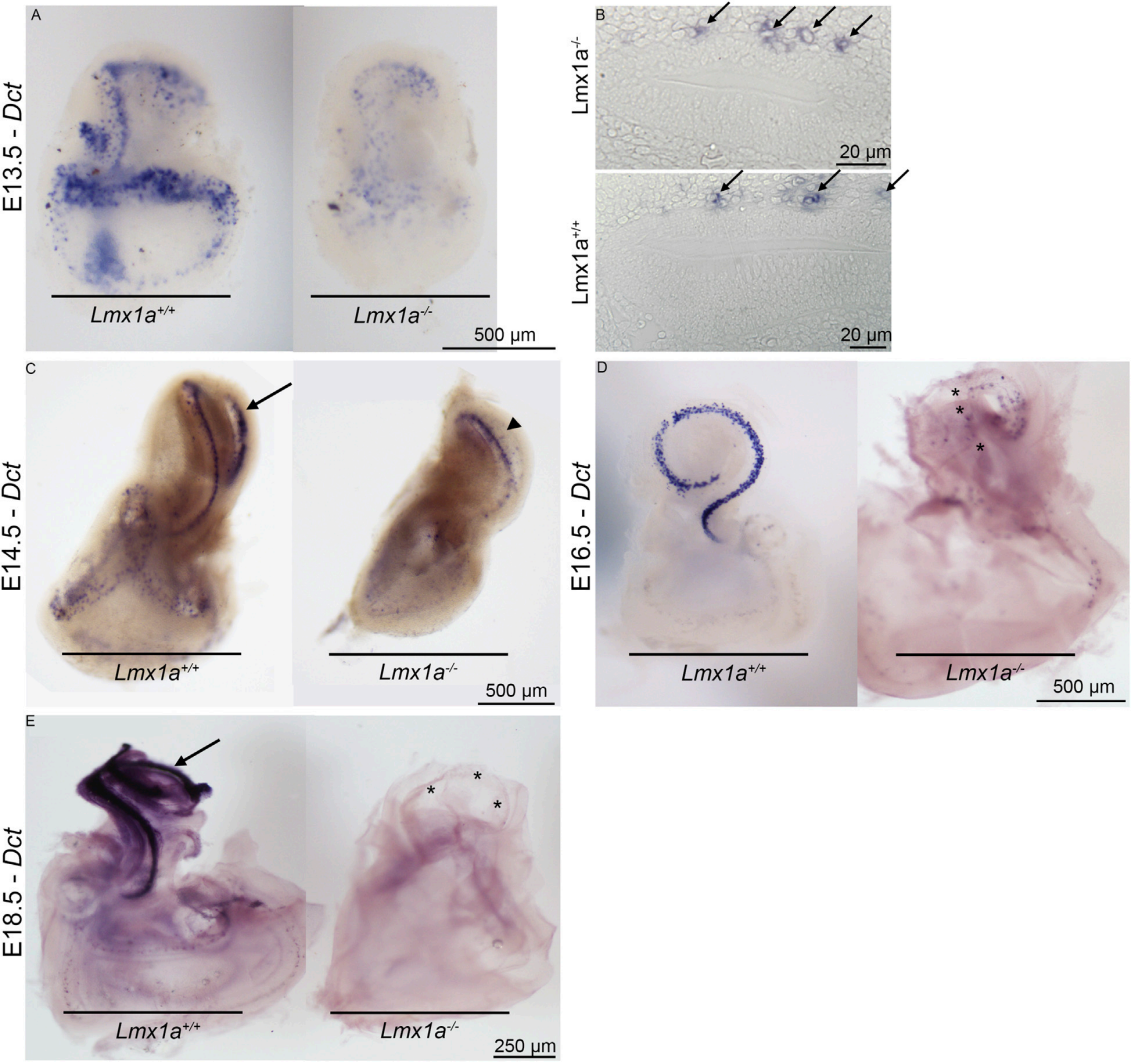
Melanoblasts are first present and then lost from *Lmx1a* ^*−/−*^ mutant ears stained for *Dct* mRNA expression. Whole-mount **(A)** and crossed section **(B)** views of E13.5 wild-type and mutant ears. Many melanoblasts have already homed to the cochlear roof regions of both (arrows). At E14.5 **(C)**, melanoblasts form a thin line following the cochlear turns (arrow). *Lmx1a* ^*−/−*^ cochlea is made of only one cochlear turn that shows labeling melanoblasts (arrowhead). In E16.5 cochlea **(D)**, most cochlear melanoblasts have homed to the pre-marginal region, showing a narrow band in the medial view of the cochlea of *Lmx1a* control mice. The cochlear cartilage was removed before staining. The intact vestibular capsule prevented staining of vestibular melanoblasts. Medial view of an E16.5 mutant ear from which the cochlear capsule and part of the vestibular capsule have been removed. Scattered melanoblasts are present, with some concentrated in the apical cochlear duct and absent in the more basal part of the cochlear turn (asterisks). Lateral views of E18.5 mutant and wild-type ears **(E)**. Cochlear capsules have been removed. Melanoblasts are abundant in the wild-type (arrow) and rare in the mutant cochlea (asterisks).

During the development of the inner ear, neural crest cells that give rise to the intermediate cell layer can be observed lining up above the roof of the cochlear duct as early as E12.5 (Renauld et al., 2022). At E15.5, the melanoblast cells start to ingress into the single-cell layer that starts differentiating into marginal cells. In the absence of *Lmx1a*, we observe the migration of melanoblasts along the membranous labyrinth from the base to the apex of the cochlear coil (E14.5 in Figures 2A–C), but 2 days later (E16.5), Dct (a melanoblast marker)-positive cells are sparse (Figure 2D, asterisk) and lost by E18.5 (Figure 2E), probably due to the absence of either intrinsic factors or signaling factors required by marginal cells in the epithelial layer of the stria vascularis. On the transverse section at E13.5, the Dct-positive cells are visible above the cochlear roof epithelium in the *Lmx1a* WT and KO mice (Figure 2B, arrows). At E14.5, *Lmx1a* ^−/−^ cochlea has one cochlear turn instead of two (Figure 2C, arrowhead versus arrow; Koo et al., (2009); Nichols et al., (2008)).

### Pigment cell abnormality in *Lmx1a* KO mice

Antibodies against several proteins can be used to label the pigment cells, including microphthalmia-associated transcription factor (MITF) and a cluster of differentiation 44 [CD44 (Renauld et al., 2022)]. We used CD44 labels, which are highly positive for pigment cells within the stria vascularis (Supplementary Figure S1B), and also labeled other cells, such as the lateral wall of the Claudius cells (CCs) (Supplementary Figure S1B′). Brainstem dye insertion shows the spiral ganglion neurons (SGNs) that radiate to reach out to the inner and outer hair cells (Supplementary Figure S1A) by radial fibers (RFs) and the modiolus. *Lmx1a* KO mice revealed poor segregation of the saccule and cochlear basal turn (Nichols et al., 2008) and showed a different pattern of radial fibers in the base (Supplementary Figures S1C, C′). Massive innervation extends to the posterior canal (PC), while the utricle (U) is incompletely fused between the anterior and horizontal canals (AC and HC). In contrast to the control mice, which show pigment cells in the stria vascularis region (Supplementary Figure S1B), *Lmx1a* KO mice exhibit a large cluster of CD44-positive cells (potentially pigment cells) that do not extend to innervate the stria vascularis (Supplementary Figures S1B, D). Similar to the WT mice, *Lmx1a* KO mice have a slight stretch of CCs labeled with CD44 (Supplementary Figures S1B′, D′).

### Otic fibrocyte and basal cell layer fail to differentiate without *Lmx1a*

**FIGURE 3:**
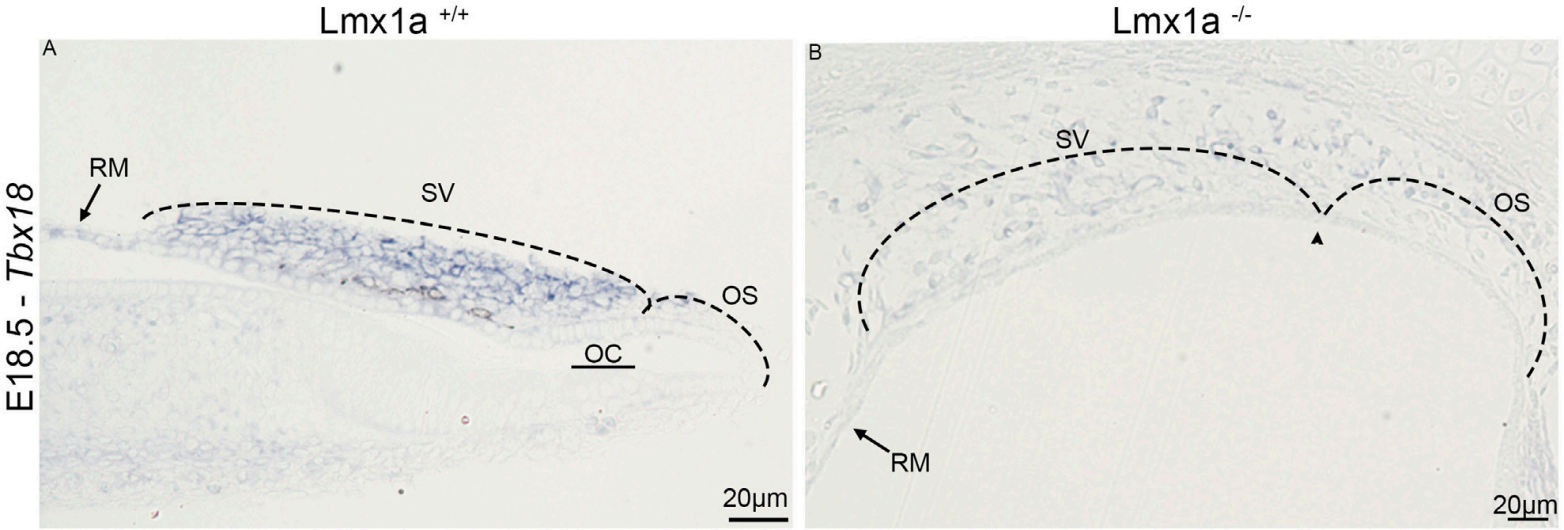
*Tbx18* mRNA expression in the periotic mesenchyme that forms the basal layer. Wild-type **(A)** and mutant **(B)** cochlear ducts stained for *Tbx18* expression. In the wild-type mice, uniformly stained cells are present beneath the pre-strial epithelium (SV), while in the mutant, they are dispersed, and separation between the outer sulcus and stria vascularis cannot be precisely determined (arrowhead). OC, organ of Corti; OS, outer sulcus; RM, Reissner’s membrane; SV, stria vascularis.

Since we observed the absence of marginal cell markers and the failure of the melanoblasts to integrate into the stria vascularis, we studied whether the mesenchymal portion of the lateral wall underneath the stria vascularis was also affected. Previous papers have shown that *Tbx18* is essential for condensation and mesenchymal–epithelial transition into the basal cell layer of the stria vascularis (Trowe et al., 2008). At E18.5, the *in situ* hybridization showed the expression of *Tbx18* in the lateral wall above the developing stria vascularis (Figure 3A). In the absence of *Lmx1a*, the expression of *Tbx18* is either exceptionally low or absent (Figure 3B). Further work using quantification is needed. Nevertheless, the near absence of *Tbx18* expression and the absence of pigment cells (unlabeled in Figure 3A) in the mesenchymal cells above the stria vascularis may explain the absence of basal layer formation, as observed in Figure 1B′.

### Expansion of pendrin expression from the spiral prominence into the location of the stria vascularis

**FIGURE 4:**
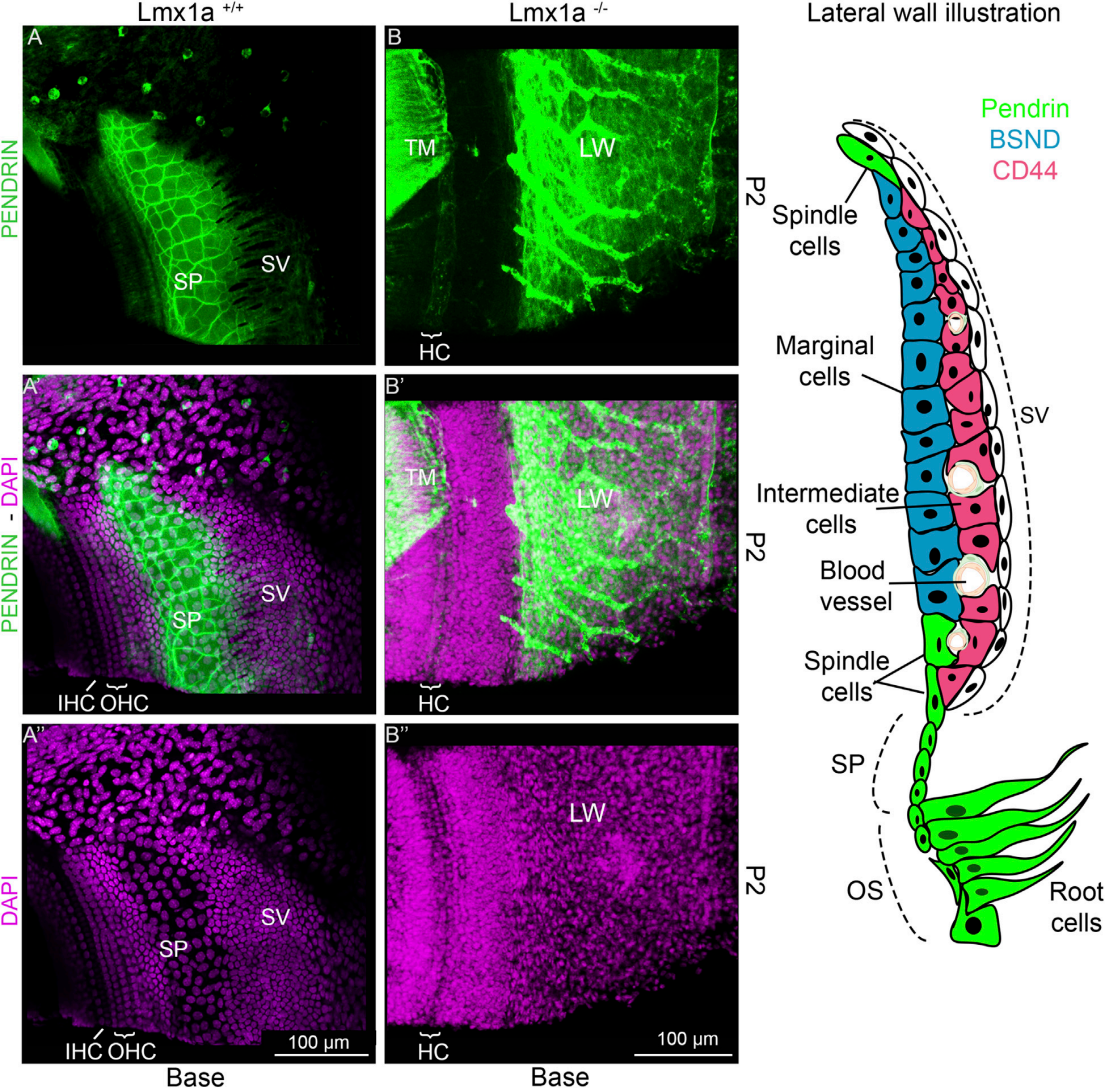
Pendrin is expressed in the spiral prominence in control and *Lmx1a* KO mice. Whole mounts flattened out of the basal tip near the ductus reuniens of the cochlea are highly positive for the pendrin antibody **(A, B)** that is expressed in the outer sulcus and spiral prominence (SP; green). Control mice **(A–Aʺ)** show that expression has a sharp definition of pendrin (green) that ends up in the base, showing the slightly larger nuclei, which are indicated adjacent to the stria vascularis (SV) that are positive for spiral prominence cells. In contrast, *Lmx1a* KO mice show no clear delineation of pendrin, which expands from the outer sulcus (OS) beyond the stria vascularis **(B–Bʺ)**. The staining of the tectorial membrane (TM) is counterstained with pendrin, which is not positive for the acellular matrix **(A, B)**. The insert on the right shows the different cell types that have been described in the stria vascularis and adjacent cells according to Koh et al. (2023). Abbreviations: CCs, Claudius cells; HCs, hair cells; LW, lateral wall; MCs, marginal cells; IHCs, inner hair cells; IMCs, intermediate cells; OHCs, outer hair cells; OS, outer sulcus; RC, root cells; SC, spindle cells; SL, spiral ligament; SP, spiral prominence. The bar indicates 100 µm.

**FIGURE 5:**
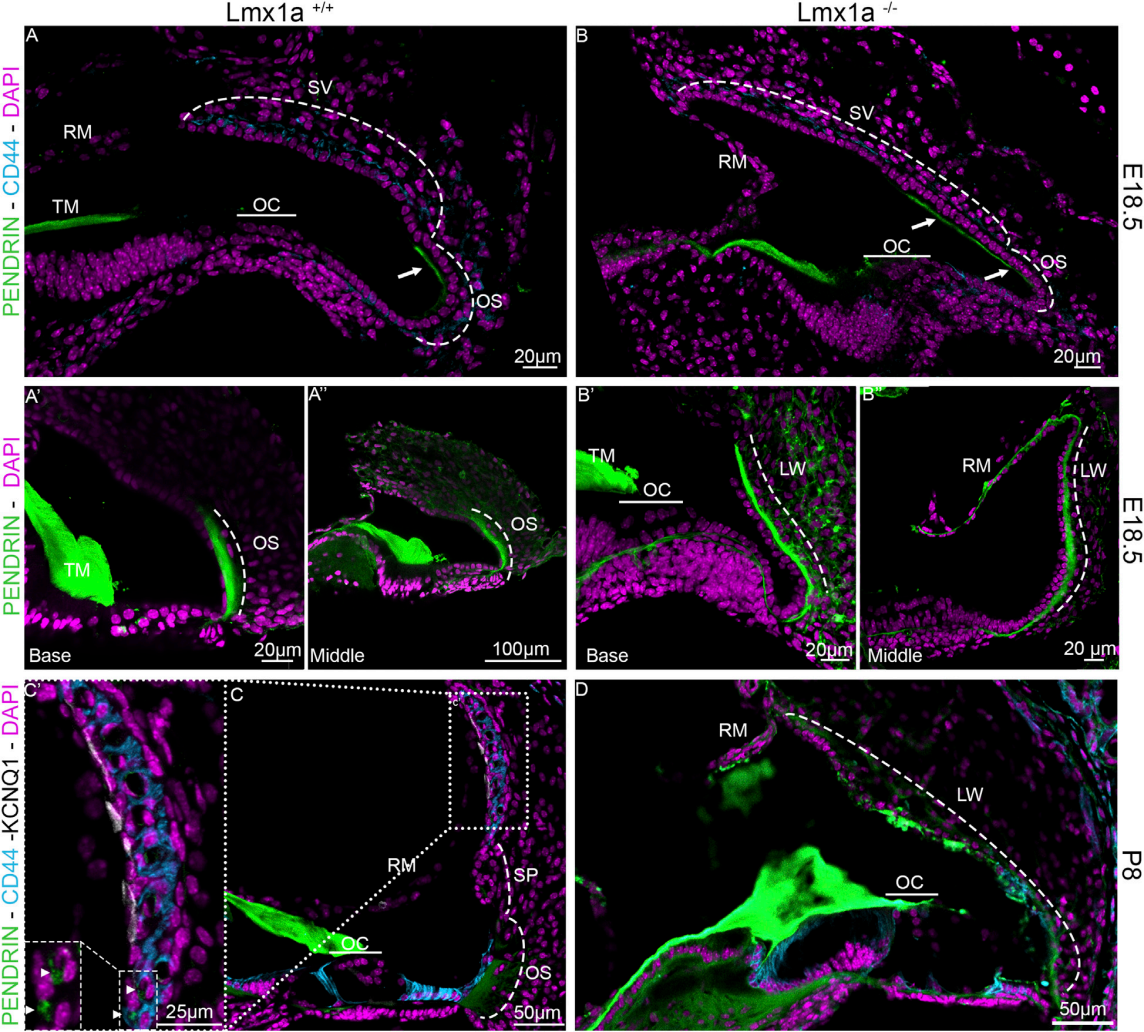
Expansion of pendrin expression in *Lmx1a* KO mice. Immunolabeling of E18.5 *Lmx1a* WT **(A)** and KO **(B)** for pendrin (green) and CD44 (cyan). At E18.5, pendrin expression is restricted to the apical portion of the outer sulcus cells **(A–Aʺ)**. In the *Lmx1a* mutant **(B–Bʺ)**, the labeling for pendrin (green) is expanded compared to WT and seems to overlap above the position of the future stria vascularis (two arrows). At P8, the stria vascularis is easily defined by marginal cells labeled by KCNQ1 (white) and intermediate cells labeled by CD44 (cyan) in *Lmx1a* ^+/+^ **(C, C′)** but not in *Lmx1a* ^−/−^ **(D)**, where pendrin (green) labels the lateral wall (LW) from the outer sulcus (OS) to Reissner’s membrane (RM; labeled with DAPI, lilac). Pendrin is also expressed in the spindle cells (arrowhead) of the stria vascularis in the WT cochlea, as shown in the magnification insert **(C′)**. Spiral prominence is indicated (SP in **(C)**).

Previous publications of *Lmx1a* KO have shown that in the absence of a functional *Lmx1a* protein, there is pendrin-positive cell expression expansion (Mann et al., 2017; Nichols et al., 2008). To verify whether this expansion changes the fate of the cells at the stria vascularis level, we analyzed the features on cochlear roof epithelium and outer sulcus cells with specific markers. In previous research, in the absence of *Esrp1*, the cochlear duct showed an expansion of Reissner’s membrane (Rohacek et al., 2017). In this study, in the absence of *Lmx1a*, we observed an expansion of the outer sulcus/spiral prominence positivity for pendrin, visible in the whole mount of the organ of Corti and lateral wall, showing intense positive staining for pendrin (Figure 4A). In control mice, a sharp positive signal contrasts with the broader and gradual reduction in pendrin expression in *Lmx1a* KO mice. Counterstaining with DAPI (Figures 4A′′, B′′) shows a reduction in the number of OHCs from three to one, adjacent to IHC (HC), as previously reported (Nichols et al., 2020; Nichols et al., 2008; Steffes et al., 2012). The density difference in the cells positive for pendrin likely corresponds to the root and the spindle cells (Figures 4A′, B′). In the whole mount, we noticed specific labeling for the acellular tectorial membrane (TM) that is, in part, removed (Figures 4A, B). The extracellular matrix of the TM can be labeled by specific and less specific labeling such as TECTA, collagen, lectin, and *Emilin2* (de Sousa Lobo Ferreira Querido et al., 2023; Fritzsch et al., 2024; Jean et al., 2023; Niazi et al., 2024). Pendrin is labeled with one antibody (Figure 4) but remains unlabeled with another antibody (Supplementary Figure S2), suggesting non-specific labeling. Overall, the expression of pendrin is not only observed in the spiral prominence but also in the root cells and outer sulcus (Figure 4A). Immunostaining on sections shows the highly positive expression of pendrin for the spiral prominence with no labeling on the stria vascularis (SV; Figures 5A–C). In contrast to pendrin-positive cells that end in the spiral prominence in the WT sections (Figures 5A–C), pendrin-positive expression continuous between the outer sulcus to the expected site for Reissner’s membrane in *Lmx1a* KO mice (Figures 5B′, B′′, D). A more detailed investigation will be required to differentiate whether it corresponds to root or spindle cells.

### *Lmx1a* is required for the development of the endocochlear potential

**FIGURE 6:**
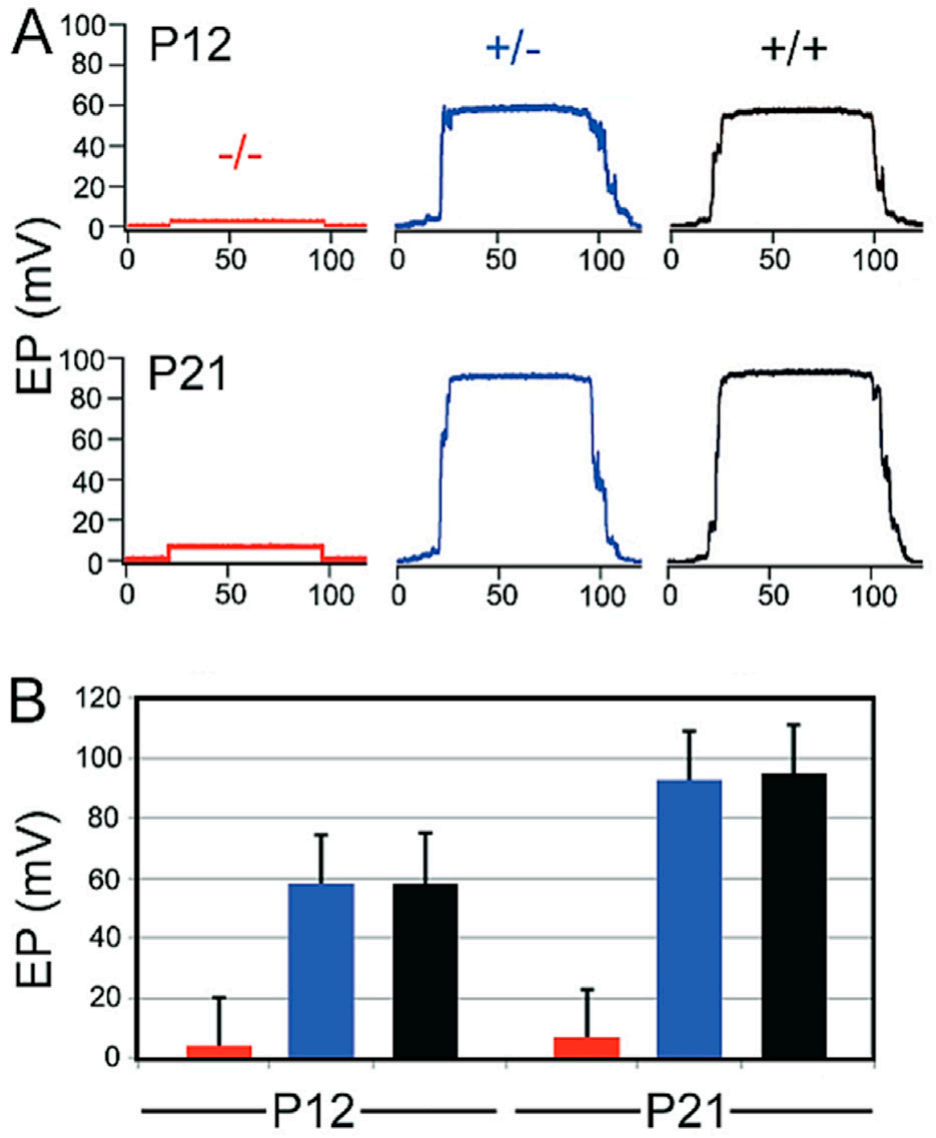
*Lmx1a* mutant never develops an endocochlear potential. Representative EP waveforms from mutant (red), heterozygous (blue), and wild-type (black) ears at P12 and P21 **(A)**. Means and standard deviations for EP’s on P12 and P21 **(B)**.

We examined the functional status of the *Lmx1a* mutant stria by comparing wild-type, heterozygote, and mutant EP (endocochlear potential) at P12 and P21. Six animals from each age/genotype were used. Figure 6 illustrates representative EP waveforms. Without a functional stria vascularis, the EP, a direct measurement of stria function, was not generated, leading to hearing loss. In this *Lmx1a* KO model, we measured the EP at P12 (during development) and P21 (mature EP) and confirmed a total absence of EP remained at less than 6-mV, as expected in the absence of a differentiated stria vascularis (Figures 6A, B). This absence of EP aligned with previous publications showing a profound hearing loss in *Lmx1a* KO mice. Thus, a functioning stria vascularis capable of producing the endocochlear potential is never present in *Lmx1a* mutant mice.

## Discussion

Our data show that the fusion of the base with the saccule in *Lmx1a* KO mice leads to an expansion of the pendrin expression in the lateral wall, which lacks a normal formation of a stria vascularis. The loss of *Lmx1a* causes the cochlear base and saccule to abnormally fuse. The ductus reuniens, which connects the cochlear base with the saccule, relies on a normal EP to maintain fluid homeostasis and separates the auditory (∼80 mV) and vestibular (∼1 mV) fluids (Figure 6).

### *Lmx1a* is required for the formation of the stria vascularis

The molecular development of the lateral wall is not fully understood, but its failure to appropriately develop leads to profound hearing loss due to its essential role in endolymph homeostasis (Bovee et al., 2024; Thulasiram et al., 2024; Wangemann, 1995). During embryonic development, the cochlear roof epithelium expresses OC90 as early as E10.5 (Hartman et al., 2015). More recently, Qin et al. showed by RNAseq that OC90-positive cells can be divided into two populations. One is the *Wnt4*-positive population, which will give rise to Reissner’s membrane, and the other is the GSC-positive population, which will give rise to the future marginal cells (Munnamalai and Fekete, 2020; Qin et al., 2024). Examples of genes essential for forming the stria vascularis have been shown through mutations, such as integrating a retrotransposon in chromosome 11 and creating a cochlear duct without a lateral wall. In this mutant, the spiral prominence attaches directly to Reissner’s membrane, creating a truncated cochlear duct, where the scala media is smaller due to the relatively low height at which Reissner’s membrane is attached (Song et al., 2021). In the knockout for a splicing protein *Espr1*, the stria vascularis is absent and replaced by a longer Reissner’s membrane due to an ectopic *Fgf9* signaling, creating a cell fate switch from marginal cells to Reissner’s membrane cells (Rohacek et al., 2017). In this study, we observed a different cell fate switch toward spiral prominence instead of Reissner’s membrane identity (Figures 1, 5). A similar cell fate has also been reported in the absence of *ERR-beta/NR3B2*, which led to a partial conversion of marginal cell fate toward the neighboring pendrin-expressing cells (Chen and Nathans, 2007). Further studies are required to elucidate the signaling pathway through which the lateral wall takes the spiral prominence identity.

During normal development, after marginal cells’ differentiation and intermediate cells’ recruitment, the mesenchymal cells aggregate and form the basal cell layer. This allows for the isolation of the stria vascularis interstitial space from the rest of the inner ear, and this separation is essential for hearing function (Gow et al., 2004; Nin et al., 2008; Souter and Forge, 1998; Wangemann, 2006; Xie et al., 2023) In the *Lmx1a* ^−/−^ mouse model, we note a single layer of epithelial cells at the level of the stria vascularis, which is visible with phalloidin staining on postnatal sections. Furthermore, the absence of the basal cell layer may be mediated by an absence of *Tbx18*, a transcription factor essential for mesenchymal aggregation onto the basal cell layer (Figure 3) during the development (Trowe et al., 2008). This absence of proper development into marginal, intermediate, and basal cell layers explains the absence of the endocochlear potential (Figure 6) and compromised hearing in *Lmx1a* KO mice, as previously reported for this mouse model (Steffes et al., 2012).

### *Lmx1a* is required for the proper recruitment of melanoblasts onto the stria vascularis

To develop properly, the stria vascularis requires the recruitment of pigment cells that will become intermediate cells and extrude K^+^ (Renauld et al., 2022; Steel and Barkway, 1989; Wangemann, 2006). In the absence of *Lmx1a*, we observed the migration of the melanoblasts (positive for *Dct* at E13.5), but later, these cells were absent from the cochlear roof (Figure 2). It would be interesting to study more systematically the disappearance of these cells to determine whether they died due to the absence of a survival signal as observed in other strial-defect-related deafness models, such as for the *MITF* mutation, where MITF has been suspected to be an important melanocyte survival factor (Ni et al., 2013; Steel and Barkway, 1989). A noticeable difference is the timeline of melanoblast disappearance occurring between P1 and P7 in the *MITF* mutation versus the current model, where the melanoblasts are almost inexistent as early as E18.5 (Figure 2E). This intrinsic defect would also explain the lack of pigmentation in the thoracic region of mutant mice (Ni et al., 2013). Another explanation for the disappearance of the melanoblasts on the roof of the cochlear duct could be that the cells integrate into the glial cells of the spiral ganglion in the absence of recruitment signals from the marginal cell layer as 80% of the intermediate cells originate from glial cell precursors (Renauld et al., 2022). Notably, many CD44-positive cells are clustered around the spiral ganglion neurons (Supplementary Figure S1), but further lineage tracing experiments are required to confirm whether these cells come from the Dct-positive cells present above the cochlear roof at E13.5.

### The absence of *Lmx1a* expands the expression of the anion exchanger protein pendrin on the lateral wall

**FIGURE 7:**
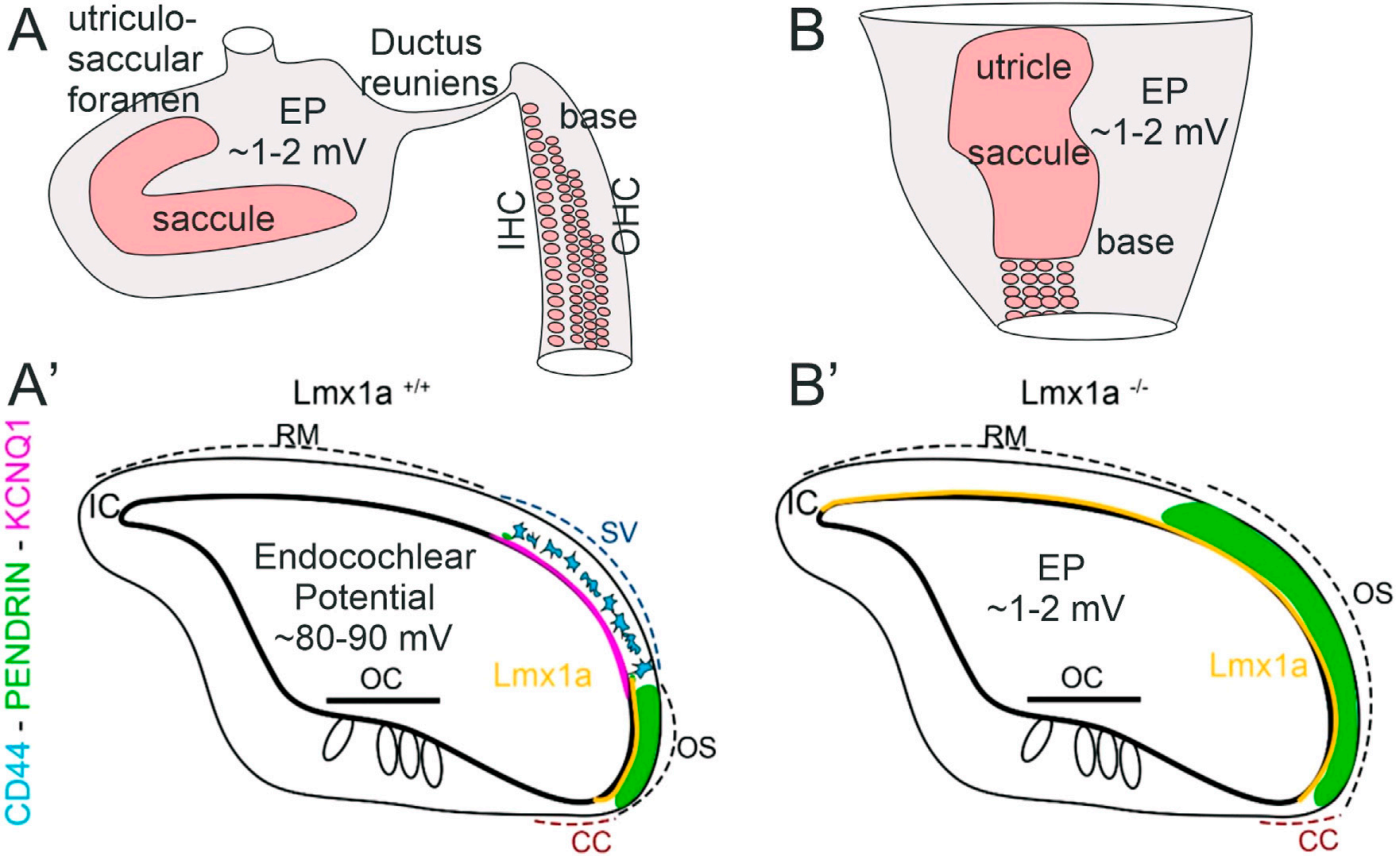
Inner ear abnormality in *Lmx1a* KO mice. In control mice **(A),** hair cell organization is nearly close to the tip of the base that segregates from the saccule by the ductus reuniens. In addition, a foramen segregates between the saccule and utricle. In contrast to *Lmx1 KO* mice **(B)**, middle turn is already broad and will be wider close to the saccule that blends with the basal tip. Note that the utricle and saccule fuse with each other and thus do not show neither the ductus reuniens nor the utriculosaccular foramen, shown in control mice. After E18.5 **(A′)**, *Lmx1a* expression is limited to the spiral prominence in WT mice adjacent to SV but expands in *Lmx1a* KO mice (Nichols et al., 2008). Our current study revealed that in *Lmx1a* KO mice **(B′)**, pendrin expression is expanded compared to wild-type mice. In WT mice, *Lmx1a in situ* signal (yellow, Nichols et al., 2008) is in the lateral wall that extends between the Claudius cells and is adjacent to the stria vascularis (SV) that contains the pigment cells that will develop into the intermediate cells (blue, CD44) adjacent to KCNQ1 (lilac). In contrast, the *Lmx1a* mutant mice show an expansion of *Lmx1a* expression. Claudius cells (CCs) and interdental cells (ICs) are weakly positive for pendrin. Neither the pigment cells (CD44 and KCNQ1) nor the stria vascularis can be found in *Lmx1a* KO mice. The pendrin protein expression (green) overlaps with *Lmx1a* in the WT but will expand and overlap with the stria vascularis in *Lmx1a* KO.

A recent analysis of the pendrin null mutation (*Slc26a4* ^*−/−*^) revealed a role of pendrin in the development of the cochlea, where it is needed for the formation of spindle cells and the spiral ligament, which contain extrinsic cellular components, a factor that enables cell-to-cell communication (Koh et al., 2023). The loss of pendrin disrupts the pH homeostasis mechanism, leading to acidic pH in the endolymphatic potential (Koh et al., 2023). As the stria vascularis is absent and pendrin expression is expanded, it would be interesting in future experiments to assess the pH present in *Lmx1a* KO mice endolymph. So far, few genes influencing pendrin have been identified in inner ear development. Pendrin expression is dependent on *Foxi1*, which regulates endolymphatic sac homeostasis and controls the hydrops formation (Hulander et al., 2003; Szeto et al., 2022). Without *Lmx1a* expression, the inner ear lacks the formation of an endolymphatic duct (Nichols et al., 2008) that is highly positive in pendrin (Figures 4, 5, 7; Supplementary Figure S2). *Foxi1* has been shown to regulate pendrin expression in the endolymphatic sac, but its absence does not prevent pendrin expression in the cochlea (Hulander et al., 2003). Pendrin is also not dependent on *Pax2*, a highly expressed transcription factor in the lateral wall of the developing cochlear duct (Bouchard et al., 2010; Burton et al., 2004; Hosoya et al., 2022). In this study, the expression of non-functional *Lmx1a* showed increased pendrin expression, extending from the spiral prominence to Reissner’s membrane (Figures 4, 5; Supplementary Figure S2). Future studies on the molecular interaction between *Lmx1a* and pendrin may be warranted.

It was shown that *SOX9* causes deafness via distinct mechanisms in the endolymphatic sac (ES)/duct and cochlea. *SOX10* is downregulated, and there is developmental persistence of progenitors, resulting in fewer mature cells. In the postnatal stria vascularis, there is impaired normal interaction of SOX9 and SOX10, repressing the expression of the water channel Aquaporin 3, thereby contributing to endolymphatic hydrops (Szeto et al., 2022). In contrast, to expand hydrops, the ear remains small in the absence of the endolymphatic sac (Nichols et al., 2008; Roux et al., 2023), which does not form a simple sac without canal cristae that continue between the saccule and basal turn in *Lmx1a* KO mice (Nichols et al., 2020).

In conclusion, the absence of *Lmx1a* profoundly alters cochlear morphology and function, preventing the formation of a functional stria vascularis and cochlear potential. This disruption leads to an abnormal auditory–vestibular continuum, shedding light on the role of *Lmx1a* in establishing the distinct ionic environments necessary for auditory and vestibular function. These findings provide important insights into congenital hearing and balance disorders linked to developmental disruptions in inner ear compartmentalization and ion homeostasis. In the absence of *Lmx1a*, we observe an absence of stria vascularis markers due to the failure of marginal cells to differentiate correctly, the inability to recruit migrating melanoblasts, and the absence of aggregation of basal cells. Furthermore, we also observed an expansion of pendrin expression (schematic, Figure 7), which highlights the importance of *Lmx1a* in the cell fate determination and differentiation of the lateral wall of the cochlear duct, which does not segregate by a ductus reuniens. Moreover, the absence of the ∼80 mV endocochlear potential in *Lmx1a* KO mice requires the three layers of the stria vascularis in the cochlea, while it forms a simpler two-layer structure in the lateral wall, resembling the vestibular system with a ∼1 mV potential.

## Data Availability

The original contributions presented in the study are included in the article/Supplementary Material, further inquiries can be directed to the corresponding authors.
