# Correction: *Lmx1a* is essential for marginal cell differentiation and stria vascularis formation

**DOI:** 10.3389/fcell.2026.1845465

**Published:** 2026-04-24

**Authors:** Justine M. Renauld, Igor Y. Iskusnykh, Ebenezer N. Yamoah, Richard J. H. Smith, Corentin Affortit, David Z. He, Huizhan Liu, David Nichols, Judith Bouma, Mahesh K. Nayak, Xin Weng, Tianli Qin, Mai Har Sham, Victor V. Chizhikov, Bernd Fritzsch

**Affiliations:** 1 Department of Biomedical Sciences, Creighton University, Omaha, NE, United States; 2 Department of Anatomy and Neurobiology, The University of Tennessee Health Science Center, Memphis, TN, United States; 3 Department of Translational Neuroscience, College of Medicine, University of Arizona, Pheonix, AZ, United States; 4 Molecular Otolaryngology and Renal Research Laboratories, University of Iowa, Iowa City, IA, United States; 5 School of Biomedical Sciences, The Chinese University of Hong Kong, Shatin, Hong Kong SAR, China; 6 Department of Neurological Sciences, University of Nebraska Medical Center, Omaha, NE, United States

**Keywords:** *Lmx1a*, pendrin, Slc26a4, stria vascularis, spiral prominence, pigment cells

An incorrect **Funding** statement was provided. The correct funding statement reads:

The author(s) declared that financial support was received for this work and/or its publication. ENY and BF were supported in part by the National Institutes of Health (grants AG060504 and AG051443 and DC016099 and DC015135); RJHS was supported in part by the National Institutes of Health (grants DC002842, DC012049, and DC017955); VC was supported by R01 NS093009 and R01 NS127973; and MHS was supported by the Research Grant Council in Hong Kong (RGC GRF 17115520). JR was supported in part by revenue from Nebraska’s Tobacco Settlement Funds through the Nebraska Department of Health and Human Services (DHHS). Its contents represent the view(s) of the author(s) and do not necessarily represent the official views of the State of Nebraska or DHHS. This research was partially conducted at the Auditory and Vestibular Technology Core (AVT) at Creighton University, Omaha, NE (RRID: SCR_023866).

The original article has been updated.

