# Supplementary material for: Lmx1a is essential for marginal cell differentiation and stria vascularis formation
Source: Front Cell Dev Biol. 2025 Mar 5;13:1537505. doi: 10.3389/fcell.2025.1537505 (PMC11920146; doi:10.3389/fcell.2025.1537505)
Supplement: Supplementary file 1 [file DataSheet1.docx]

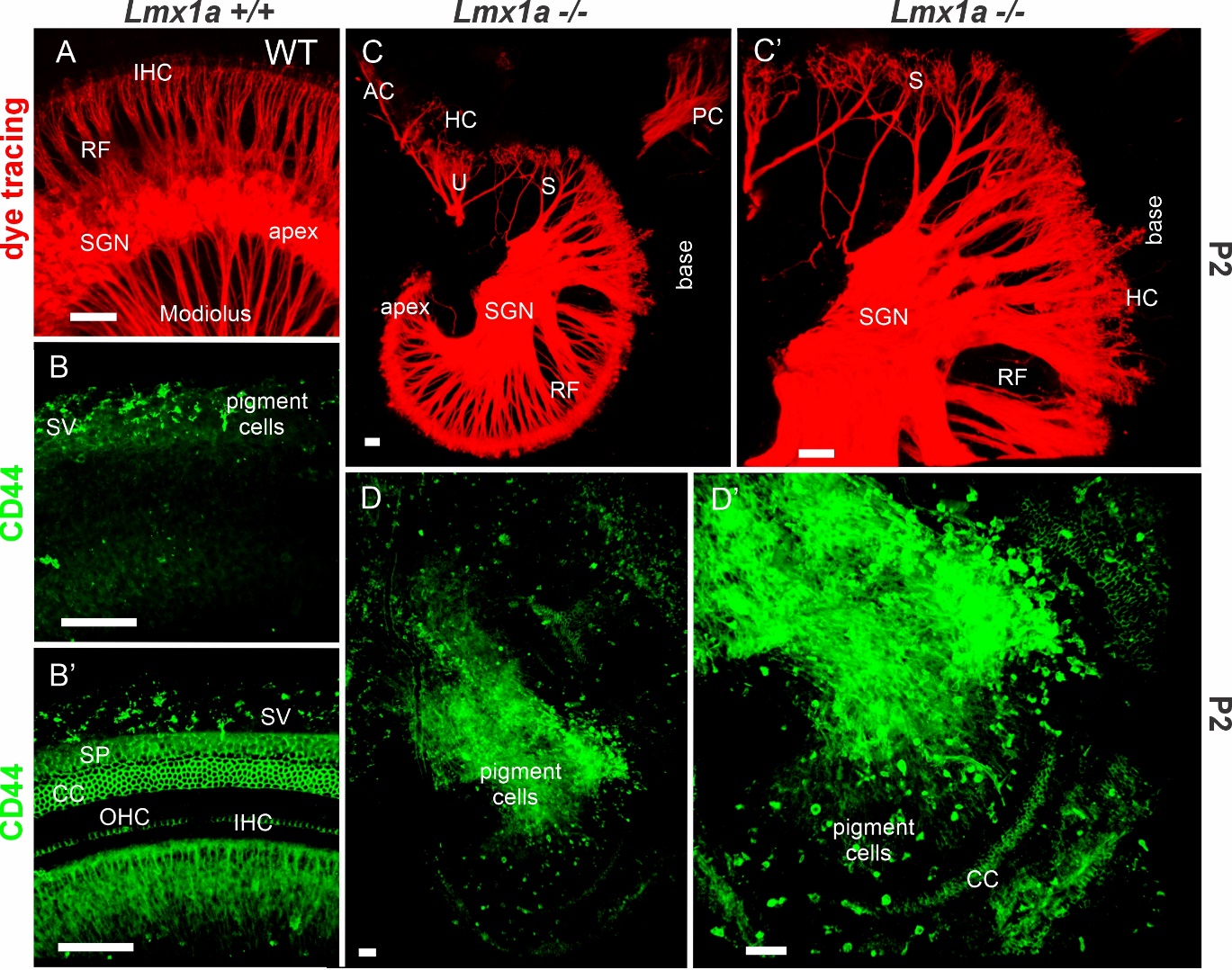


**Supplement Fig. S1**: Pigment cell pattern differs in *Lmx1a* KO mice. As a background, we labeled with dye tracing to show the afferent nerve pattern in a 2-day-old mouse (P2, A, C). Instead of separating a ductus reuniens between the saccule and the basal turn, it fuses with the saccule/basal turn (C, C’). Whole-mount high-resolution CD44 staining (B, B’) demonstrates the unique positive pigment cells. At a lower level, we show the Claudius cells (CC) are located adjacent to the spiral prominence (SP) near the stria vascularis (SV). The formation of pillar cells (not labeled) separates the inner and outer hair cells (IHC, OHC). The SGNs split at the base with a mix of cochlear and vestibular hair cells (C, C’) while the apex follows the normal pattern of hair cell distribution. A significant difference between control (B) and *Lmx1a* KO mice is the clustering of pigment cells near the SGNs (D’D’), while control mice have a unique localization of pigment cells essential for the formation of the stria vascularis. Moreover, the absence of pigment cells affects other proteins, which are a short stretch of Claudius cells (B’, D’) that are medial to the CC, while in control mice, they are migrating beyond the spiral ligament (B’). Bars indicates 100 µm.


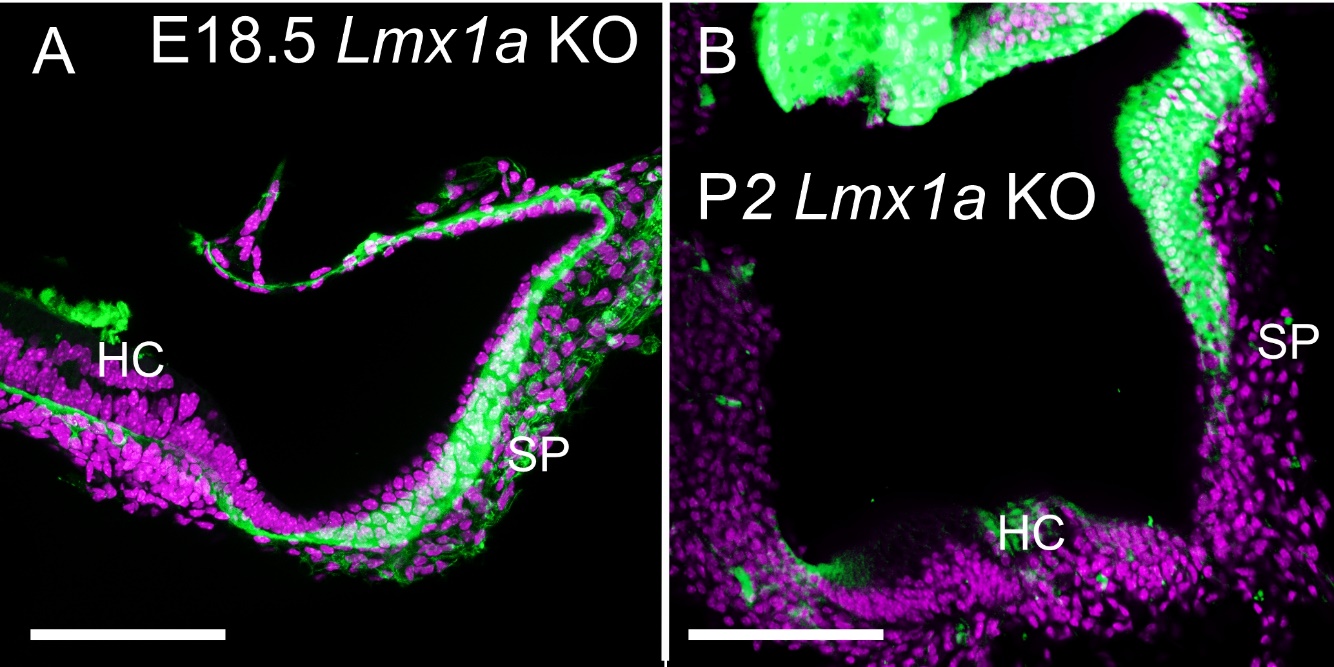


**Supplement Fig. S2:** Pendrin immunostaining (green) using an antibody characterized by (Roux et al., 2023). Similar to commercially available Pendrin antibody (Koh et al., 2023), in Lmx1a KO mice, this antibody shows Pendrin staining that stretches from the spiral prominence (SP) beyond the stria vascularis. HC, hair cells. Bars indicates 100 µm.
